# Physiotherapeutic Protocol Focusing Proprioceptive Neuromuscular Facilitation Approach in Viral Encephalitis: A Case Report

**DOI:** 10.7759/cureus.47636

**Published:** 2023-10-25

**Authors:** Vaishnavi M Thakre, Snehal Samal, Devyani Purushe

**Affiliations:** 1 Neuro-Physiotherapy, Ravi Nair Physiotherapy College, Datta Meghe Institute of Higher Education and Research Institute of Medical Sciences, Wardha, IND

**Keywords:** pnf, varicella zoster virus, case report, physiotherapeutic rehabilitation, encephalitis

## Abstract

Encephalitis is an inflammatory condition of the brain parenchyma accompanied by symptoms indicative of brain dysfunction. A headache, fever, vomiting, altered state of consciousness, and, in some cases, seizures characterize varicella-zoster virus (VZV) encephalitis. Rehabilitation after the manifestation of neurological symptoms must be tailored, and a well-coordinated intervention must be formulated. The proprioceptive neuromuscular facilitation (PNF) technique is a widespread rehabilitation approach used to restore motor function. A 29-year-old male is presented in this case report with VZV viral encephalitis with complaints of generalized weakness, headache, and oromotor difficulties. This case report specifies the physiotherapeutic protocol, mainly focusing on the PNF approach. Patient’s occupational requirements and enhancement in executing daily living tasks were the focus of the physiotherapeutic rehabilitation. The outcomes used were the Berg Balance Scale (BBS) and Functional Independence Measure (FIM). We report a marked increment in muscle tone and strength, active range of motion (AROM), and significant enhancement in the individual’s functional independence with physiotherapeutic protocol post-operatively.

## Introduction

Encephalitis is characterized by brain parenchymal inflammation with associated localized or generalized neurological impairment [1,2]. It is characterized by symptoms such as headache, fever, and a change in the level of consciousness. An emergence of generalized or focal seizure activity can occur as well [3]. Various infectious agents, including bacteria and viruses, cause encephalitis [4]. Herpes simplex virus (HSV) is the most commonly found pathogen, followed by varicella-zoster virus (VZV), an enterovirus that causes viral encephalitis. Bacterial infections are most usually caused by* Mycobacterium tuberculosis* and *Listeria monocytogenes* [5]. Infectious causes continue to constitute the majority of cases with a known etiology; however, autoimmune causes are becoming more common. Type-A gamma-aminobutyric acid (GABAa) receptor antibodies have recently been identified in encephalitis with refractory seizures [6,7].

In broad terms, encephalitis should be speculated when signs and symptoms of neurologic dysfunction which include altered level of consciousness, behavioural changes, headache, seizures, papilledema, and focal impairment, alongside systemic manifestations such as respiratory symptoms, fever, rash, lymphadenopathy, myalgia, arthralgia or gastrointestinal tract (GIT)-related symptoms, or when there is an exposure history to known risk factors (insects or ticks, travelling to endemic areas, animal bites) [8,9]. Diagnostic methods include brain imaging, generally non-contrasted computed tomography (CT) of the brain. Magnetic resonance imaging (MRI) may help in the differential diagnosis of idiopathic inflammatory CNS illnesses by better illustrating brain inflammation and demonstrating focal lesions [10]. Electroencephalography (EEG) should be done on all patients to identify generalized or focal epileptiform activity. In certain circumstances, EEG can assist in distinguishing whether behavioural abnormalities are solely psychological or caused by underlying encephalopathy [11].

Proprioceptive Neuromuscular Facilitation (PNF) is a technique that involves stimulating proprioceptors in various tissues, including the joints, skin, tendons, and muscles. This stimulation is utilized to optimize neuromuscular processes such as muscular strength, endurance, mobility, joint stability, neuromuscular control, and balance [12,13]. For building muscular strength and flexibility, various methods such as rhythmic initiation, repetitive contractions, rhythmic stabilization, a combination of isotonics, dynamic reversals, hold-relax, and contract-relax can be implemented [14]. There is currently a lack of studies demonstrating a specific approach or optimal technique for applying PNF to improve motor function in patients with encephalitis. As a result, we established a training programme based on the PNF approach to help these patients improve their motor control, functional independence and gait.

## Case presentation

Patient information

A 29-year-old male, working as a driver, was brought to Acharya Vinoba Bhave Rural Hospital (AVBRH) unconscious. The patient was stable until August 16, 2023, when a sudden loss of consciousness occurred, followed by the onset of initial convulsions. On the same day, the patient also experienced an episode of acute fever. He regained consciousness for a brief period of 15 minutes, only to lose consciousness again. The patient had undergone certain investigations like an MRI and was diagnosed with viral encephalitis. Presently, the patient complains of generalized weakness, headache, and oromotor difficulties. He reported a history of alcoholism spanning eight years. Additionally, there was a documented history of reduced appetite and weight loss over the past 15 days. He did not present any known comorbidities or complaints related to bladder or bowel function. On August 18, 2023, physiotherapy rehabilitation was initiated, following a carefully tailored protocol designed for the patient.

Clinical findings

Prior to commencing the examination, the patient's informed consent was obtained, following which a thorough examination was conducted. During the examination, the patient exhibited cooperation but also displayed lethargy. Remarkably, he remained well-oriented to person, place, and time. The patient had a normal body temperature and stable hemodynamics. He assumed a supine position with a 30-degree elevation of the head end and appropriate support for the knees and ankles using pillows. Physically, the patient presented an ectomorphic physique, evidenced by a BMI of 19 kg/m². Although his speech was altered, his vision and hearing were within normal parameters. The neurological evaluation confirmed intact sensations. However, there was a reduction in both tone and muscle strength, as outlined in Table 1, observed in both upper and lower limbs. Notably, all deep tendon reflexes demonstrated diminished activity, as detailed in Table 2. The presence of a positive Babinski's sign was noted, and the patient exhibited an inability to stand and walk. Assessment using the Functional Independence Measure (FIM) indicated that the patient requires maximal assistance for basic Activities of Daily Living (ADLs) encompassing bathing, eating, toileting, and transferring. Additionally, the patient necessitates maximal assistance for instrumental ADLs such as transportation, communication, and medication handling.

**Table 1:** Pre-intervention muscle strength on the Manual Muscle Testing (MMT) scale 0: No contraction; 1: Flickering contraction; 2: Full Range of Motion (ROM) with gravity eliminated; 3: Full ROM against gravity; 4: Full ROM against gravity, moderate resistance; 5: Full ROM against gravity, maximal resistance

Muscles	Right	Left
Shoulder Flexors	1/5	1/5
Shoulder Extensors	1/5	1/5
Shoulder Abductors	1/5	1/5
Elbow Flexors	1/5	1/5
Hip Flexors	1/5	1/5
Hip Extensors	1/5	1/5
Hip Abductors	1/5	1/5
Hip Adductors	1/5	1/5
Knee Flexors	1/5	1/5
Knee Extensors	1/5	1/5

**Table 2:** Pre-intervention deep tendon reflexes +: Diminished reflex, ++: Normal reflex, +++: Exaggerated reflex

Reflexes	Right	Left
Bicep jerk	+	+
Tricep jerk	+	+
Knee jerk	+	+
Ankle jerk	+	+
Plantar reflex	Extensor	Extensor

Diagnostic assessment

**Figure 1:**
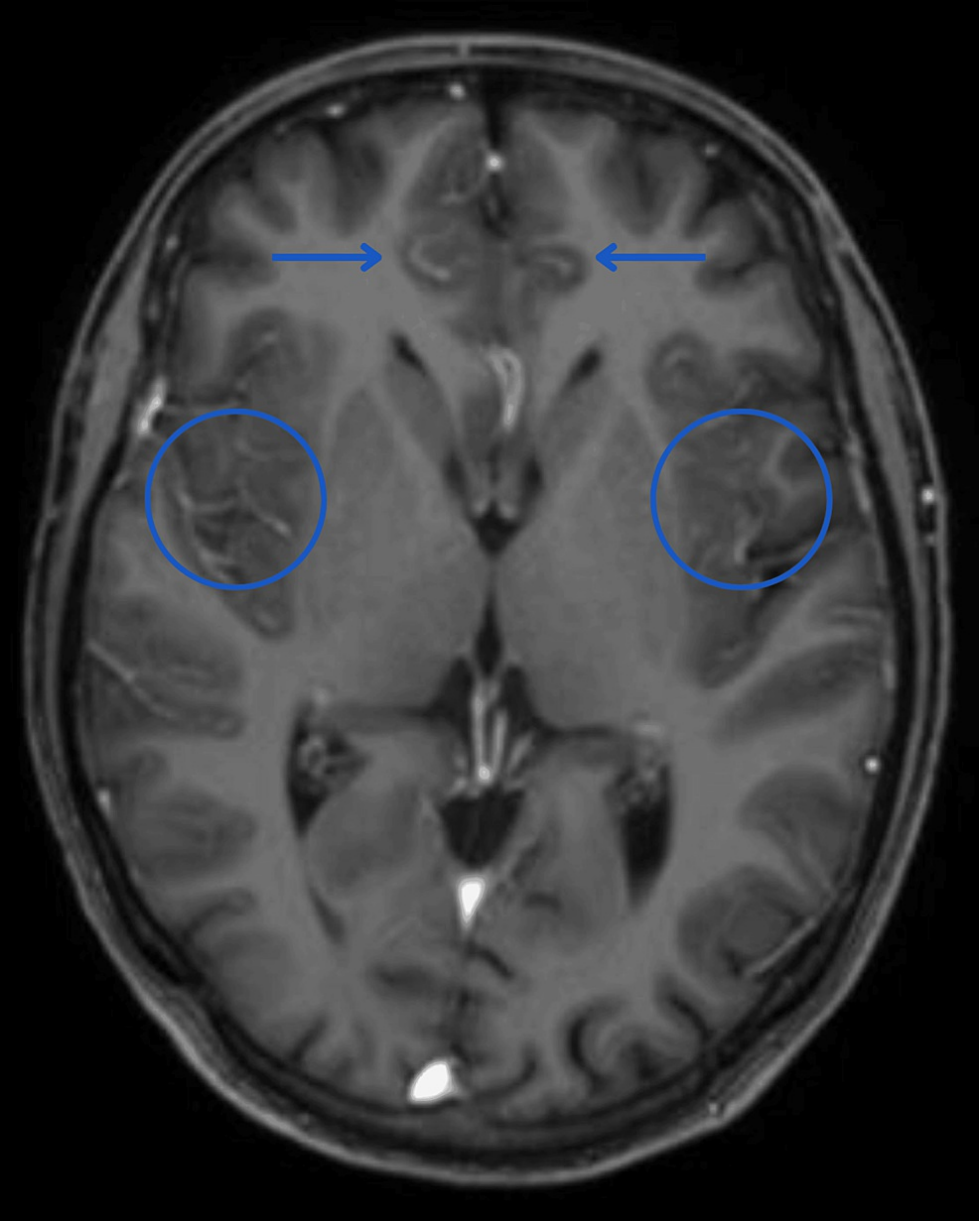
MRI-Brain revealing acute hyperammonemic or acute hepatic encephalopathy MRI: Magnetic Resonance Imaging Blue arrows point to the non-enhancing symmetrical signal abnormalities with diffusion restriction in the bilateral parasagittal frontal region involving the cingulate gyrus. Blue circles reveal non-enhancing symmetrical signal abnormalities with diffusion restriction in the bilateral parasagittal frontal region involving the bilateral insular cortex.

A complete blood count (CBC) and cerebrospinal fluid (CSF) examination were done. In these investigations, increases in levels of white blood cells and C-reactive protein were found. The MRI-Brain was performed as a diagnostic tool, which revealed non-enhancing symmetrical signal abnormalities with diffusion restriction in the bilateral parasagittal frontal region involving the cingulate gyrus, bilateral insular cortex, and bilateral temporal region. The above features favour a diagnosis of acute hyperammonemic or acute hepatic encephalopathy rather than an ischemic event (Figure 1).

Timeline of the current episode

In August 2023, the patient was admitted to the neurology ward. On day 3, he was referred for physiotherapeutic management. The patient received treatment for four weeks.

Physiotherapy intervention

**Figure 2:**
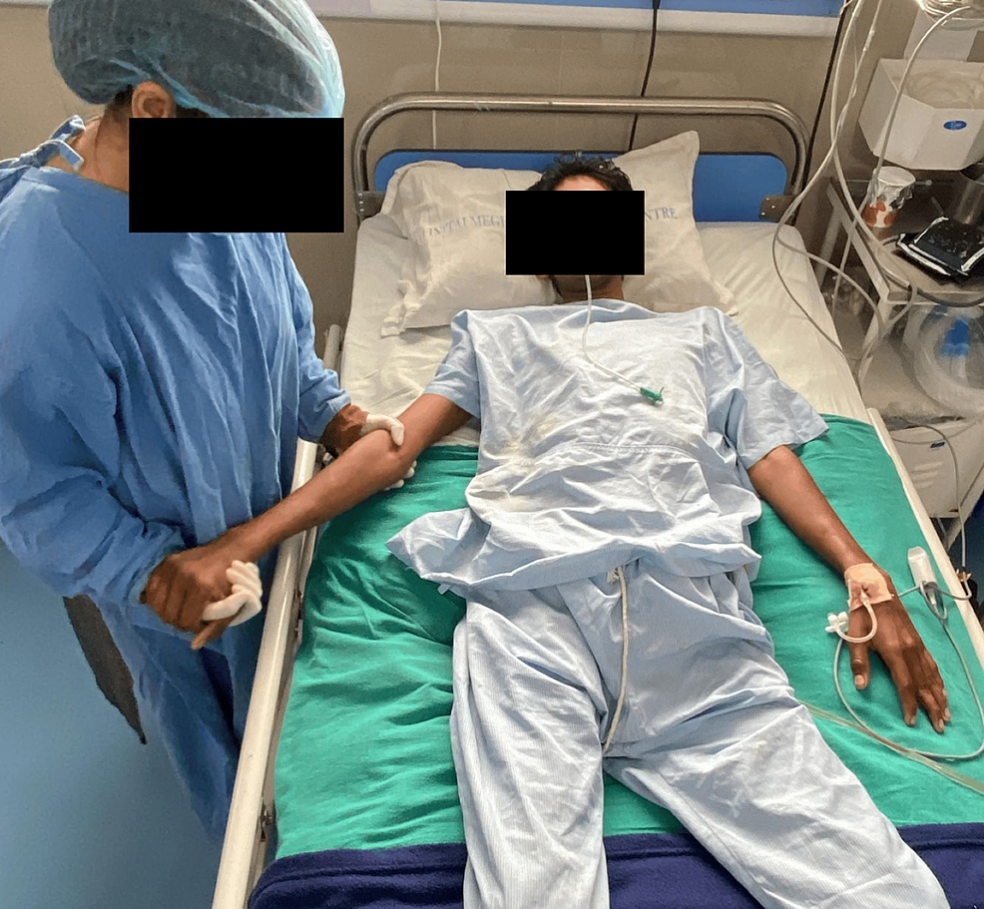
Upper limb (right) PNF rhythmic initiation PNF: Proprioceptive Neuromuscular Facilitation

**Figure 3:**
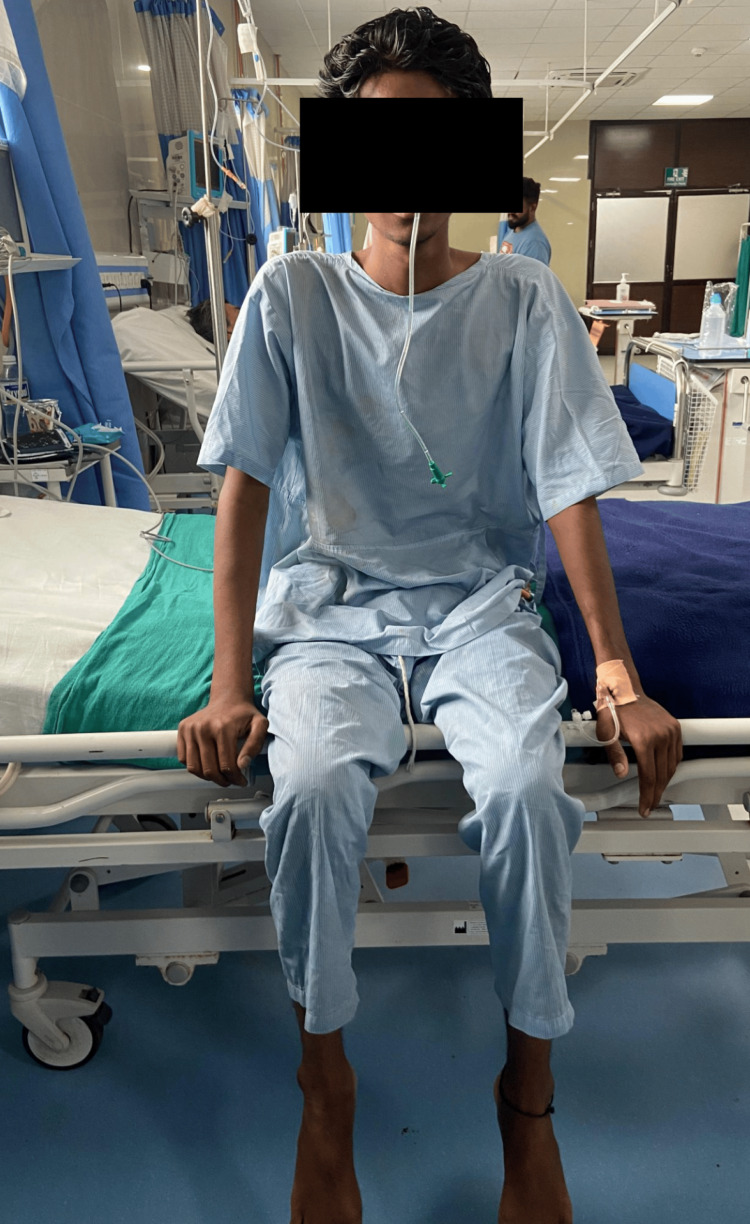
Patient sitting independently

**Figure 4:**
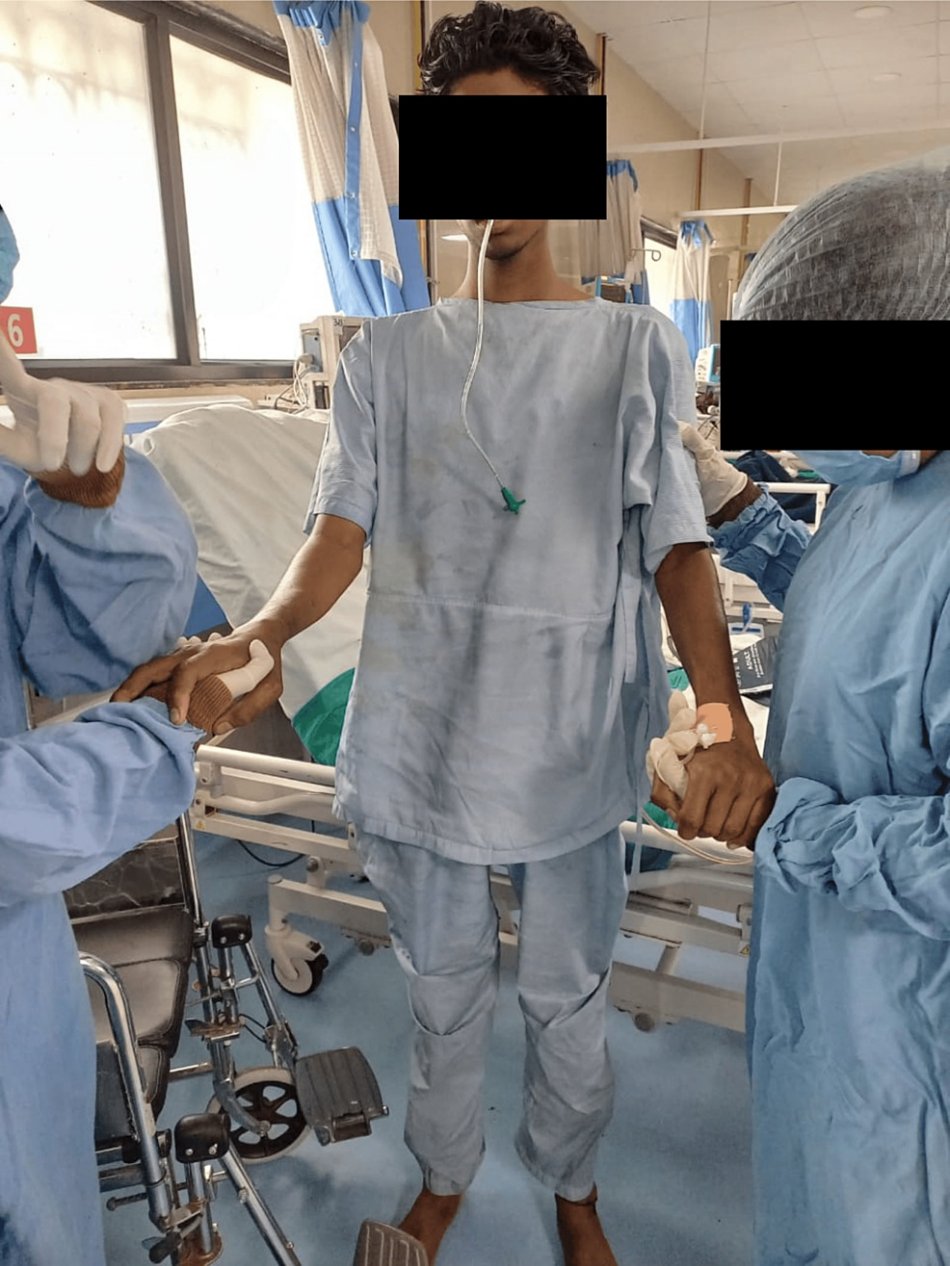
PNF sit-to-stand training PNF: Proprioceptive Neuromuscular Facilitation

**Figure 5:**
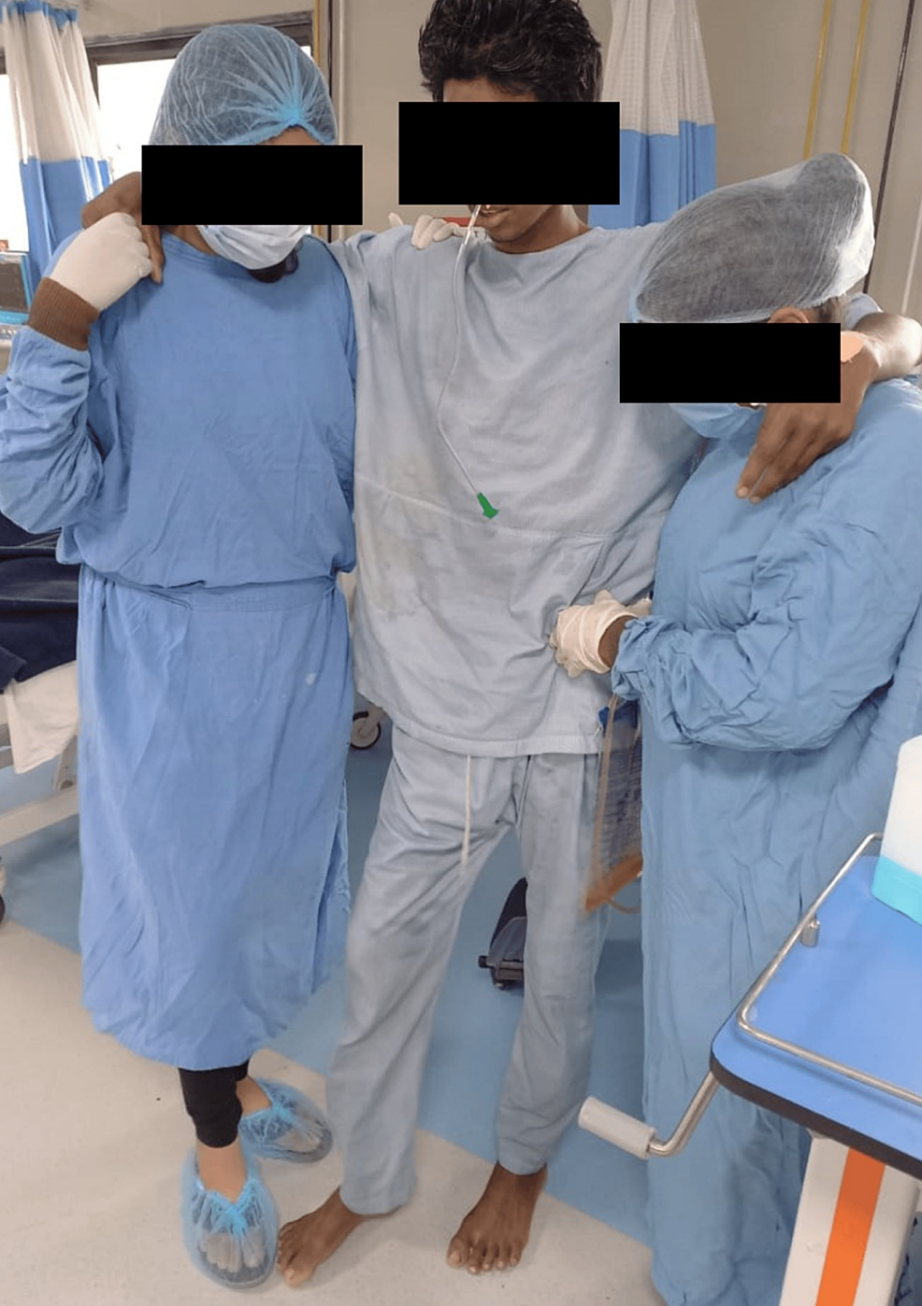
PNF gait training (weight shifting and stepping strategies) PNF: Proprioceptive Neuromuscular Facilitation

Physiotherapy (PT) rehabilitation protocol was planned week-wise (Table 3). Physiotherapy intervention received by the patient is summarized in Figures 2-5. Figure 2 shows the therapist giving the PNF rhythmic initiation technique to the right upper limb. In Figure 3, the patient has gained independent sitting balance. Figures 4-5 reveal the progression of the patient from standing to initiating ambulation.

**Table 3:** Physiotherapy intervention N/A: Not applicable, PNF: Proprioceptive Neuromuscular Facilitation, ROM: Range of motion, reps: repetitions

Problem identified	Goal	Treatment Strategy	Intervention	Progression
1) Patient and Family Education	To enhance and maintain patient’s positive attitude towards treatment for early recovery.	Interaction of therapist involving patient and his family.	Patient and along with his family was well-explained regarding his condition and was told about the importance of physiotherapy intervention.	The home programme was given and explained.
Hypotonia	To enhance muscular tone	Facilitation techniques for tone development	PNF Rhythmic Initiation for upper and lower limb (D1 and D2 patterns) (10 reps x 1 set) (Figure 2)	PNF slow reversals and combination of isotonics (10 reps x 1 set)
			Joint approximation (10 rep x 1 set)	
Unable to perform bed mobility independently	To promote bed mobility.	Functional mobility activities	Mat Exercises	N/A
Reduced muscle strength	To increase muscular strength of both upper and lower limb	Resistance exercises with PNF.	PNF contract-relax for upper and lower limb (10 reps x 1 set)	PNF hold-relax for upper and lower limb (10 reps x 1 set)
			Strengthening exercises with ½ kg weight cuff	Strengthening exercises with 1 kg weight cuff
Inefficient trunk and pelvic control	To improve trunk and pelvic control	Core Stability exercises	Pelvic PNF	Pelvic muscle strengthening on therapy ball (5 minutes)
			Pelvic bridging exercises (10 reps with 5 seconds hold x 1 set)	
			Half push-ups in prone lying (6 reps x 1 set)	Full push-ups in prone lying (10 reps x 1 set)
Balance and ambulation impairment	To gain static and dynamic balance	Balance and ambulation retraining	PNF Sit to Stand training (Figure 3 and 4)	PNF Gait training (weight shifting and stepping strategies) (Figure 5)
Lack of deglutition and speech	To improvise deglutition and speech	Oro-motor retraining	Jaw exercises	Strengthening of muscles of mastication
			Stroking (using light brush)	
			Lip exercises	
			Tongue range of motion (ROM) exercises	

Followup and outcomes

An organized physical therapy interventional protocol was started. For four weeks, a follow-up was carried out once per week. The findings of the outcome measure are shown in (Tables 4-6).

**Table 4:** Pre and post physiotherapeutic rehabilitation muscular strength MMT: Manual muscle testing, 0: No contraction; 1: Flickering contraction; 2: Full Range of Motion (ROM) with gravity eliminated; 3: Full ROM against gravity; 4: Full ROM against gravity, moderate resistance; 5: Full ROM against gravity, maximal resistance

Muscle Group	Pre-treatment MMT grade	Post-treatment MMT grade
Shoulder Flexors	1/5	4/5
Shulder Extensors	1/5	4/5
Shoulder Abductors	1/5	4/5
Elbow Flexors	1/5	4/5
Hip Flexors	1/5	4/5
Hip Extensors	1/5	4/5
Hip Abductors	1/5	4/5
Hip Adductors	1/5	4/5
Knee Flexors	1/5	5/5
Knee Extensors	1/5	5/5

**Table 5:** Pre and post physiotherapeutic rehabilitation tone (TGS) TGS: Tone Grading Scale; 1+: Decreased tone, 2+: Normal tone, 3+: Increased tone

Muscle Group	Pre-intervention	Post-intervention
Shoulder Flexors	1+	2+
Shulder Extensors	1+	2+
Shoulder Abductors	1+	2+
Elbow Flexors	1+	2+
Hip Flexors	1+	2+
Hip Extensors	1+	2+
Hip Abductors	1+	2+
Hip Adductors	1+	2+
Knee Flexors	1+	2+
Knee Extensors	1+	2+

**Table 6:** Pre and post physiotherapeutic rehabilitation outcome measures

Scale	1^st^ week	4^th^ week
Functional Independence Measure	18/126	70/126
Berg Balance Scale	7/56	36/56

## Discussion

In the clinical setting, encephalitis is not rare. It should be addressed whenever clinical signs and investigations are related to other prevalent causes of encephalitis, such as infections and toxins. Prompt diagnosis and intensive therapy provide positive results.

There are no formal guidelines for physiotherapy programmes for individuals having encephalitis. There is a lack of neuroscientific research on the positive impacts of physiotherapy protocol on individuals suffering from this disease. A few studies on the beneficial effect of physical rehabilitation on the patient’s functioning, conducted in an interdisciplinary team with other professionals, are available [15,16]. Physiotherapy encompasses exercises to improve limb function and trunk stabilization. Passive exercises on limbs were carried out to avoid muscle contractures and preserve joint range of motion. Active exercises for the lower limb and orofacial treatment were also incorporated. Comprehensive physiotherapy substantially enhanced the individual's communication ability along with social life engagement. Communication methods for those with substantial motor impairments, including speech disorders, continue to be a challenge for medicine, necessitating customized implementation and selection based on the patient's functional skills in conjunction with rehabilitation [17].

The Swiss ball has proven to be an effective tool for strengthening trunk core muscles, improving trunk muscular activity, achieving upright posture stability, and providing upper limb flexibility of movement. The Swiss ball, unlike other adaptive aids such as wobble board, Bosu ball, and foam mats, commences weight-shifting with minimal transitions, seeking less therapist effort. This procedure is more effective than usual therapeutic strategies [18].

According to Cayco et al., a proprioceptive neuromuscular facilitation approach combined with neuroplasticity principles was reliable and efficient for restoring the motor outputs of elderly people having chronic stroke [19]. Selective PNF approaches can improve movement accuracy and speed, particularly in non-spastic muscle groups [20].

## Conclusions

With the global rise in viral encephalitis cases, conducting thorough and effective studies on diverse treatment modalities is crucial. This case study underscores the significance of a well-structured four-week physiotherapeutic intervention complemented by the PNF approach, showcasing enhanced treatment outcomes. It emphasizes the remarkable benefits of this approach in facilitating motor relearning, improving muscle tone and strength, addressing balance and gait impairments, enhancing overall quality of life, and promoting functional independence for patients dealing with viral encephalitis.
